# The novel Lyme borreliosis vaccine VLA15 shows broad protection against *Borrelia* species expressing six different OspA serotypes

**DOI:** 10.1371/journal.pone.0184357

**Published:** 2017-09-01

**Authors:** Pär Comstedt, Wolfgang Schüler, Andreas Meinke, Urban Lundberg

**Affiliations:** Valneva Austria GmbH, Vienna, Austria; University of Kentucky College of Medicine, UNITED STATES

## Abstract

We have previously shown that the Outer surface protein A (OspA) based Lyme borreliosis vaccine VLA15 induces protective immunity in mice. Herein, we report the induction of protective immunity by VLA15 with mouse models using ticks infected with *B*. *burgdorferi* (OspA serotype 1), *B*. *afzelii* (OspA serotype 2) and *B*. *bavariensis* (OspA serotype 4) or with *in vitro* grown *B*. *garinii* (OspA serotype 5 and 6) for challenge. For *B*. *garinii* (OspA serotype 3), we have developed a growth inhibition assay using chicken complement and functional antibodies targeting *B*. *garinii* (OspA serotype 3) could be demonstrated after immunization with VLA15. Furthermore, following three priming immunizations, a booster dose was administered five months later and the induction of immunological memory could be confirmed. Thus, the antibody titers after the booster dose were increased considerably compared to those after primary immunization. In addition, the half-lives of anti-OspA serotype specific antibodies after administration of the booster immunization were longer than after primary immunization. Taken together, we could show that VLA15 induced protection in mice against challenge with four different clinically relevant *Borrelia* species (*B*. *burgdorferi*, *B*. *afzelii*, *B*. *garinii* and *B*. *bavariensis*) expressing five of the six OspA serotypes included in the vaccine. The protection data is supported by functional assays showing efficacy against spirochetes expressing any of the six OspA serotypes (1 to 6). To our knowledge, this is the first time a Lyme borreliosis vaccine has been able to demonstrate such broad protection in preclinical studies. These new data provide further promise for the clinical development of VLA15 and supports our efforts to provide a new Lyme borreliosis vaccine available for global use.

## Introduction

Lyme borreliosis (LB) is an emerging infectious disease transmitted by ticks in the northern hemisphere. Although several attempts to develop a prophylactic vaccine have been made, no vaccine for human use is available today [1–4]. Two different human LB Outer surface protein A (OspA) based vaccines have demonstrated efficacy in clinical trials; LYMErix (SmithKline Beecham) and ImuLyme (Pasteur Mérieux-Connaught) for the US. In addition, a multivalent OspA vaccine (Baxter Bioscience) for Europe has been tested in Phase I/II clinical trials [1–3]. However, none is currently in use. LYMErix was licensed in the US from 1998 to 2002, but was voluntarily withdrawn from the market [5]. We have previously published an approach for the rational design of a new OspA based LB vaccine (VLA15) targeting the clinically most relevant *Borrelia* species and OspA serotypes (ST) present in Europe and the US, namely *B*. *burgdorferi* (ST1), *B*. *afzelii* (ST2), *B*. *garinii* (ST3, ST5 and ST6) and *B*. *bavariensis* (ST4) [6]. The VLA15 vaccine is based on the notion that the C-terminal part of OspA is sufficient to induce protective immunity [7]. Therefore, by using the C-terminal part of six OspA serotypes (ST1 to ST6) stabilized with disulfide bonds, and linking two monomers together in each of the three fusion proteins, we have generated a new LB vaccine for global use [6]. Furthermore, introducing a lipid moiety at the N-terminus of each fusion protein and formulating the vaccine with aluminium hydroxide strongly increased the immunogenicity in mice [6]. The vaccine induced a protective immune response against challenge with *in vitro* grown *B*. *burgdorferi* (ST1) or *B*. *garinii* (ST5) as well as with ticks infected with *B*. *afzelii* (ST2) [6]. We could also demonstrate the induction of a functional immune response with surface binding for all OspA serotypes and growth inhibition assays for five of the six OspA serotypes included in the vaccine [6]. In order to improve the OspA ST3 specific immunogenicity as well as the yield of the fusion protein representing OspA ST3 and ST4, a modified protein was designed [8]. In the new fusion protein, referred to as Lip-D4Bva3B, approximately 1/3 of the N-terminal part of the OspA ST3-monomer has been exchanged with the corresponding sequence of OspA from *B*. *valaisiana*. The new heterodimer had an significantly improved expression and purification profile as well as increased OspA ST3 specific immunogenicity [8]. The new formulation includes the three fusion proteins Lip-D1B2B, Lip-D4Bva3B and Lip-D5B6B in a 1:1:1 ratio and is referred to as VLA15 [8]. Further details describing the concept, design and characterization of VLA15 were described previously [6,8].

Other OspA based vaccines have been assessed for protection in mice following challenge with different *Borrelia* species. The studies have used either *in vitro* grown *B*. *burgdorferi* (ST1) [9,10], *B*. *garinii* (ST5) [6] or *B*. *garinii* (ST6) [11]. Alternatively using laboratory reared ticks infected with either *B*. *burgdorferi* (ST1) [12] or *B*. *afzelii* (ST2) [6,13]. We have now been able to assess the efficacy of VLA15 following challenge with *Borrelia* species expressing five different OspA serotypes; *B*. *burgdorferi* (ST1), *B*. *afzelii* (ST2), *B*. *bavariensis* (ST4) or *B*. *garinii* (ST5 and ST6). Protection against the first three mentioned *Borrelia* species was assessed in challenge models where the natural vector, *Ixodes* ticks, were used for challenge of VLA15 immunized mice. Tick challenge models for *B*. *garinii* (ST3, ST5 and ST6) have thus far not been described. Growth inhibition assays were described for the OspA serotypes 1, 2, 4, 5 and 6 by us [6,8] and others [3]. However, a functional assay with *B*. *garinii* ST3 has still been missing, likely because the spirochetes were sensitive to the guinea pig complement alone, and this source of complement was commonly used in the assay as established by Sadziene and coworkers [14]. By further development of our growth inhibition assay, we were able to study the bactericidal effect of anti-VLA15 immune sera with regards to *B*. *garinii* (ST3).

OspA is expressed in culture and on the spirochete surface when in the tick gut. It is down regulated once the tick begins to feed and is replaced by OspC on the surface. Anti-OspA antibodies act in the tick gut to block transmission. Therefore, protection is dependent on a sufficient level of circulating anti-OspA specific antibodies. In order to avoid frequent booster immunizations, a strong and long-lasting immune response is desirable [15,16]. In 2002, at the time when LYMErix was withdrawn from the market, it was not known how long a protective immune response would last. Therefore, it was not determined at what interval booster immunization would be required [5,17]. LYMErix was administered with a schedule of 0, 1 and 12 months. One month after the third immunization, subjects had a GMT of 6,000 enzyme immunoassay units (EIA U)/mL and 90% of those had a titer ≥1,400 which ensures protection over one tick season. Nearly all subjects that got infected had an anti-OspA antibody titer ≤400 EIA U/mL at the time of LB onset [18,19]. Therefore, we assessed the antibody titers induced by VLA15 in mice for one year and determined the effect of a booster immunization administered five months after the primary immunization.

In this article we demonstrate exceptionally broad protection in several novel mouse models following VLA15 immunization and challenge with infected ticks or *in vitro* grown spirochetes unsurpassed by any preceding LB vaccine [20–22]. In addition, data describing the induction of long lasting immunological memory and the effect of a booster immunization further support the development of the VLA15 vaccine which has recently entered clinical evaluation.

## Materials and methods

### Ethics statement and animal care

All animal experiments were conducted in accordance with Austrian law (BGBl No. 114/2012) and approved by ‘‘Magistratsabteilung 58”. Experimental procedures were reviewed and approved by Valneva’s animal welfare committee and experiments performed by trained personnel that all have successfully completed a FELASA (Federation of Laboratory Animal Science Association) B course. The animals were housed at the Valneva Austria GmbH animal facility in Vienna at 20–24°C with a 12/12 hours light/dark cycle in standard IVC cages (Euro standard Type II long) in groups of five female mice in each cage. Mice were fed a commercial mouse chow (Ssniff RIM-H autoclaved, Ssniff Spezialdiäten GmbH, Germany) ad libitum and had free access to tap water. Food and water were autoclaved before they were given to the mice. As environmental enrichment mice were provided wood wool as nesting material. All animals kept in the animal facility of Valneva Austria GmbH in Vienna are checked daily. When an animal shows clinical signs the responsible veterinarian is immediately informed, the veterinarian checks the animal and decides which measures that has to be taken. No mortalities occurred prior to the conclusion of the experiments and were also not expected, since mice are the natural reservoir of *Borrelia* in nature.

Animals were anaesthetized by an intraperitoneal injection with 0.3 mL of a 10% Ketamine (Ketamidor, Richter Pharma AG) and 3% Xylazine (Rompun, Bayer Healthcare) mixture when the ventilated containers were mounted. When ticks were applied to the mice and for the collection of terminal blood Isoflurane (Baxter Healthcare) was administered using a HNG-6 Anesthesia machine (H.Holzel Laboratory Equipment GmbH, German). Every effort was made to minimize animal suffering.

### *Borrelia* strains used and infection of ticks with *B*. *burgdorferi* and *B*. *bavariensis*

The *Borrelia* strains used in this study, *B*. *garinii* strains PFe, PFr and Z10 (ST3), PHei (ST5) and KL11 (ST6), have been kindly provided by Dr. Volker Fingerle (German National Reference Centre for Borrelia, Bavarian Health and Food Safety Authority).

In order to infect laboratory reared ticks with new *Borrelia* strains, feral ticks were collected from two different biotopes in Vienna, Austria (the public park Prater [48.2013, 16.4256]) and St. Marx cemetery [48.18236, 16.40187] in the park areas without grave yards), no permission was needed for the collection of ticks in public areas. DNA was extracted from a subset of the ticks and analyzed with qPCR targeting *ospA* to determine the infection prevalence and the OspA serotype of the infecting *Borrelia* strains. Remaining ticks were fed individually on naïve C3H/HeN mice (Janvier, France). Four weeks later, the infection status of mice was determined by VlsE ELISA and qPCR (targeting *ospA*), using a serum sample and a skin biopsy collected from one ear, respectively. Positive samples were subjected to *ospA* sequencing to determine the OspA serotype of the infecting *Borrelia* species [6], mice infected with spirochetes expressing only one OspA serotype were kept, mice infected with spirochetes expressing different OspA serotypes were not kept for further analyses. Mice infected with *B*. *burgdorferi* (ST1, strain Pra1 from a tick collected in the public park Prater [48.20104, 16.42486]), *B*. *bavariensis* (ST4, Marx1 from a tick collected in the St. Marx cemetery) or *B*. *bavariensis* (ST4, Marx2 from a tick collected in the St. Marx cemetery [48.18257, 16.40195]) were infested with naïve larvae (Insect Services, Berlin). The mice were individually housed in cages with a grid floor having a tray with water underneath where the fed larvae from each mouse were collected. The larvae were allowed to molt and the nymphs derived from each mouse were used for subsequent infection of two gerbils (Insect Services). The following two to six weeks, larvae were fed on the infected gerbils and let to molt. The nymphs from the gerbils infected with the same strain were randomized and used for challenge of immunized mice as described elsewhere [6].

### Vaccine efficacy studies in mice

All animal experiments were conducted as earlier described [6]. In short, six to eight-weeks-old female C3H/HeN mice (Janvier) were used for all studies. All vaccines were formulated with 0.15% aluminium hydroxide (Alhydrogel®, Brenntag, Denmark). Groups of ten mice were immunized subcutaneously (s.c.) three times with 100 μL vaccine at two-weeks interval. Four different doses of VLA15 were tested; 3.0, 0.3, 0.03 and 0.003 μg. One week after the third immunization, blood was collected and sera were prepared. The mice were challenged two weeks after the last immunization with *I*. *ricinus* infected with *B*. *afzelii* (ST2) strain IS1 (Insect Services), *B*. *burgdorferi* (ST1) strain Pra1 or *B*. *bavariensis* (ST4) strain Marx1. The tick challenge followed the same procedures as earlier described [6]. Alternatively, mice were challenged s.c. with 1×10^4^ *in vitro* grown *B*. *garinii* (ST5) strain PHei, or 1×10^2^ *in vitro* grown *B*. *garinii* (ST6) strain KL11 [23,24]. The *in vitro* grown spirochetes were analyzed by flow cytometry and challenge of mice was only performed with cultures where >80% of spirochetes were positive for OspA surface expression. Four (challenge with *in vitro* grown spirochetes) or six weeks (challenge with spirochete infected ticks) after the challenge blood was collected by orbital bleeding and sera prepared. Mice were sacrificed by cervical dislocation and the urinary bladder and both ears were collected and subjected to DNA extraction. The infection status of mice was determined by VlsE ELISA and qPCR targeting *recA* (nucleotide 334 to 524) [6,25]. In the passive immunization experiments, mice were injected intraperitoneally with 200 μL serum, one day later blood was collected and sera were prepared. Subsequently on the same day, mice were challenged as described above, with the exception that *I*. *ricinus* ticks were used infected with *B*. *bavariensis* (ST4) strain Marx2 instead of strain Marx1.

### Long term immunogenicity study

The immunization of mice followed the same procedure as described for the vaccine efficacy studies with a few exceptions. The immunization dose was 3.0, 0.3 or 0.03 μg VLA15 formulated with 0.15% aluminium hydroxide. A booster immunization was administered 5 months after the third priming immunization and mice were kept for another 6 months before they were sacrificed. Following the third priming immunization, blood was collected every fourth week for the whole study period of one year and immune sera prepared. The immune response was determined by endpoint ELISA using pooled immune sera from 10 mice and plates coated with full-length OspA ST1—ST6 [6].

### Endpoint ELISA

The OspA ELISA was performed as described before [6]. In short, ELISA plates were coated with full-length OspA in PBS and incubated at 4°C overnight. The plates were blocked with blocking buffer (PBS with 1% BSA, 0.05% Tween® 20) for 1–2 hours at room temperature. Pooled sera from 10 mice were diluted in blocking buffer (five-fold dilution) and tested in duplicates. The secondary antibody (horseradish peroxide conjugated rabbit anti-mouse IgG, DAKO, Denmark) was diluted 1:2,000 in blocking buffer and incubated for 1 hour at room temperature. ABTS (Sigma-Aldrich) was used as substrate for horseradish peroxide, the reaction was stopped after 15 min by the addition of 1% SDS and the absorbance was read at 405 nm. The endpoint titer is defined as the reciprocal of the highest serum dilution giving a reading above the cutoff (set as three times the mean background absorbance).

### Growth inhibition assay

The growth inhibition assay was performed as earlier described [6], but with modifications for *B*. *garinii* (ST3). Since all *B*. *garinii* (ST3) strains tested were sensitive to guinea pig, mouse and human complement, active complement from birds was finally used (Agrisera, Sweden). The complement was prepared from freshly collected chicken blood, by letting it clot for 20 min followed by separation of the sera by centrifugation in a table top centrifuge at 2,200 rpm for 8 min. Aliquots of active complement were directly frozen and stored at -80°C until further use. The growth inhibition activity in sera from mice immunized with either 3 μg VLA15 or 1 μg His-tagged and lipidated full-length OspA ST3 (Lip-OspA3-His) was compared. Briefly, serial dilutions (1:5) of heat-inactivated immune sera were incubated with spirochetes (1×10^4^ cells) in the presence of 1% chicken complement for four days at 35°C and 1% CO_2_. The number of spirochetes was determined by flow cytometry [6].

## Results

### Establishing tick colonies for OspA serotypes 1 and 4

By collecting ticks from different areas endemic for Lyme borreliosis in Vienna (Austria) and allow them to feed on naïve mice, we have successfully established tick colonies with a *B*. *burgdorferi* (ST1) strain (Pra1, from the public park Prater in Vienna) and two *B*. *bavariensis* (ST4) strains (Marx1 and Marx2, from St. Marx cemetery in Vienna). Attempts to establish tick colonies with *B*. *garinii* OspA ST3, ST5 and ST6 infected ticks have so far not been successful, while a stable transmission between ticks and mice could not be established, usually the infection was lost in the ticks during molting to nymphs. To our knowledge, colonies with *B*. *garinii* (ST3, ST5 and ST6) infected ticks have so far never been reported.

### Active immunization with VLA15 demonstrates protection against five OspA serotypes

The preclinical studies to assess the protective efficacy after active immunization with VLA15, composed of the three fusion proteins Lip-D1B2B, Lip-D4Bva3B and Lip-D5B6B in a 1:1:1 ratio formulated with 0.15% aluminium hydroxide [8], were performed using female C3H/HeN mice. Groups of ten mice were immunized three times with VLA15 and challenged with nymphal *I*. *ricinus* ticks infected with *B*. *burgdorferi* (ST1, strain Pra1), *B*. *afzelii* (ST2, strain IS1) or *B*. *bavariensis* (ST4, strain Marx1). Alternatively, mice were challenged subcutaneously with *in vitro* grown *B*. *garinii* (ST5, strain PHei) or *B*. *garinii* (ST6, strain KL11) [23,24]. The complexity of the tick challenge model only allows for a limited number of mice to be included in each experiment (see materials and methods section for details). Therefore, all VLA15 immunization doses could not be tested in one and the same experiment. For this reason, we have combined data from different experiments where the same lot of vaccine and batch of infected ticks were used. However, all experiments followed an identical protocol and were performed by the same technical staff.

VLA15 induced significant protective immunity at the lowest immunization dose tested, 0.003 μg, against challenge with ticks harboring *B*. *afzelii* (ST2) or *B*. *bavariensis* (ST4) (Table 1). Furthermore, an immunization dose of 0.03 μg provided protection against challenge with *in vitro* grown *B*. *garinii* (ST5) (Table 1). An immunization dose of 0.3 μg was needed to generate protection against challenge with *B*. *burgdorferi* (ST1) transmitted by infected ticks (Table 1). Some level of protection was also observed in the two lowest dose groups (0.03 and 0.003 μg) after challenge with *B*. *burgdorferi* (ST1), albeit without reaching statistical significance.

**Table 1:** Efficacy of VLA15 after active immunization of mice and challenge either with ticks infected with *B*. *burgdorferi* (ST1), *B*. *afzelii* (ST2) or *B*. *bavariensis* (ST4) or *in vitro* grown *B*. *garinii* (ST5 or ST6).

Immunization		Infected/Total (percentage infected mice)					
		Tick challenge			*in vitro* grown spirochetes		
		*B*.* burgdorferi*	*B*.* afzelii*	*B*.* bavariensis*	*B*.* garinii*		*B*.* garinii*
Immunogen	Dose	Pra1 (ST1)	IS1 (ST2)	Marx1 (ST4)	PHei (ST5)		KL11 (ST6)
VLA15	3 μg	1/13*** (8%)	1/16*** (6%)	0/11*** (0%)	0/10*** (0%)	1/10*** (10%)	3/10** (30%)
	0.3 μg	1/21*** (5%)	0/15*** (0%)	0/15*** (0%)	0/10*** (0%)	0/10*** (0%)	nd
	0.03 μg	8/20^ns^ (40%)	3/16*** (19%)	0/13*** (0%)	2/10* (20%)	3/10** (30%)	nd
	0.003 μg	12/21^ns^ (57%)	8/14* (57%)	4/11^ns^ (36%)	7/10^ns^ (70%)	7/9^ns^ (78%)	nd
Placebo	-	13/19 (68%)	17/17 (100%)	12/15 (80%)	8/10 (80%)	9/9 (100%)	10/10 (100%)

Mice were immunized subcutaneously three times with a two week interval and challenged two weeks after the third immunization. Ticks infected with *B*. *burgdorferi* (ST1, strain Pra1), *B*. *afzelii* (ST2, strain IS1) or *B*. *bavariensis* (ST4, strain Marx1) were used for challenge. Only mice with at least one fully or almost fully fed tick (≥48 hours feeding) were included in the readout. Due to the complexity of the tick challenge model, all immunization doses could not be tested in one experiment. Therefore, data from three (*B*. *burgdorferi*) or two (*B*. *bavariensis* and *B*. *afzelii*) separate experiments respectively, have been combined. All experiments used the same batch of ticks infected with corresponding *Borrelia* species for challenge, followed an identical protocol and were performed by the same technical staff. Alternatively, immunized mice were challenged with 1×10^4^ *in vitro* grown *B*. *garinii* (ST5, strain PHei) or 1×10^2^ *B*. *garinii* (ST6, strain KL11). P-values were calculated with Fisher’s exact test (two tailed)

* <0.05,

** <0.01 and

*** <0.001 and

^ns^ not significant. nd—not done.

We have tested three *B*. *garinii* (ST3) strains (PFe, PFr and Z10) which express OspA on their surface and are virulent in mice. However, when mice were immunized with 1 μg full length lipidated OspA (ST3, Lip-OspA3-His) formulated with 0.15% aluminium hydroxide and challenged with any of these three strains, no significant protection were observed when compared to the placebo group. Thus, we have so far not been able to perform vaccine efficacy studies in mice with VLA15 against *B*. *garinii* (ST3).

### VLA15-induced antibodies provide protection against five OspA serotypes upon passive immunization

In addition to active immunization, we have also assessed protection provided after passive immunization with pooled immune sera from mice immunized three times with two week intervals, with 5.0 μg VLA15 formulated with 0.15% aluminium hydroxide. Protection was tested in the same mouse models as described for the active immunization studies. Mice were passively immunized intraperitoneally with 200 μL serum at varying dilutions of the immune serum (Table 2). The immune serum was diluted with a serum pool from naïve mice. Mice were challenged on the following day, either with spirochete infected ticks (*B*. *burgdorferi* (ST1, strain Pra1), *B*. *afzelii* (ST2, strain IS1) or *B*. *bavariensis* (ST4, strain Marx2)) or with *in vitro* grown spirochetes (*B*. *garinii* (ST5, strain PHei) or *B*. *garinii* (ST6, strain KL11)) as described in material and methods.

**Table 2:** Efficacy of VLA15 after passive immunization of mice and challenge either with ticks infected with *B*. *burgdorferi* (ST1), *B*. *afzelii* (ST2) or *B*. *bavariensis* (ST4) or *in vitro* grown *B*. *garinii* (ST5 or ST6).

Immunization		Infected/Total (percent infected mice)					
		Tick challenge			*in vitro* grown spirochetes		
		*B*.* burgdorferi*	*B*.* afzelii*	*B*.* bavariensis*	*B*.* garinii*		*B*.* garinii*
Immunogen	Dose	Pra1 (ST1)	IS1 (ST2)	Marx2 (ST4)	PHei (ST5)		KL11 (ST6)
VLA15	200 μL	1/16*** (6%)	1/19*** (5%)	1/8** (12%)	0/10*** (0%)	0/10*** (0%)	0/10** (0%)
	140 μL	0/18*** (0%)	3/18*** (17%)	0/2^ns^ (0%)	0/10*** (0%)	1/10*** (10%)	1/10* (10%)
	80 μL	1/16*** (6%)	6/19*** (32%)	0/6** (0%)	0/10*** (0%)	3/10^ns^ (30%)	2/10 ^ns^ (20%)
	20 μL	9/19^ns^ (47%)	12/19** (63%)	2/5^ns^ (40%)	2/10^ns^ (20%)	4/10^ns^ (40%)	1/10* (10%)
Placebo	-	11/17 (65%)	20/20 (100%)	6/6 (100%)	7/10 (70%)	8/10 (80%)	7/10 (70%)

Mice were immunized intraperitoneally the day before challenge. Ticks infected with *B*. *burgdorferi* (ST1, strain Pra1), *B*. *afzelii* (ST2, strain IS1) or *B*. *bavariensis* (ST4, strain Marx2) were used for challenge. Data from two separate experiments for *B*. *burgdorferi* (ST1, strain Pra1) and *B*. *afzelii* (ST2, strain IS1) have been combined. Alternatively, 1×10^4^ *in vitro* grown *B*. *garinii* (ST5, strain PHei) or 1×10^2^ *B*. *garinii* (ST6, strain KL11) were used for challenge. P-values were calculated with Fisher’s exact test (two tailed)

* <0.05

** <0.01 and

*** <0.001 and

^ns^ not significant. For further information see legend to Table 1.

Mice were protected against challenge using infected ticks and *in vitro* grown spirochetes, and a dose-dependent protection against challenge with spirochetes expressing any of the five OspA serotypes was observed (Table 2). The lowest immunization dose (20 μL immune serum) was sufficient to induce significant protection against challenge with *B*. *afzelii* (ST2) or *B*. *garinii* (ST6). Furthermore, the second lowest immunization dose (80 μL immune sera) protected mice against challenge with any of the other strains: *B*. *burgdorferi* (ST1), *B*. *bavariensis* (ST4) or *B*. *garinii* (ST5).

### A growth inhibition assay for *B*. *garinii* (ST3)

**Fig 1:**
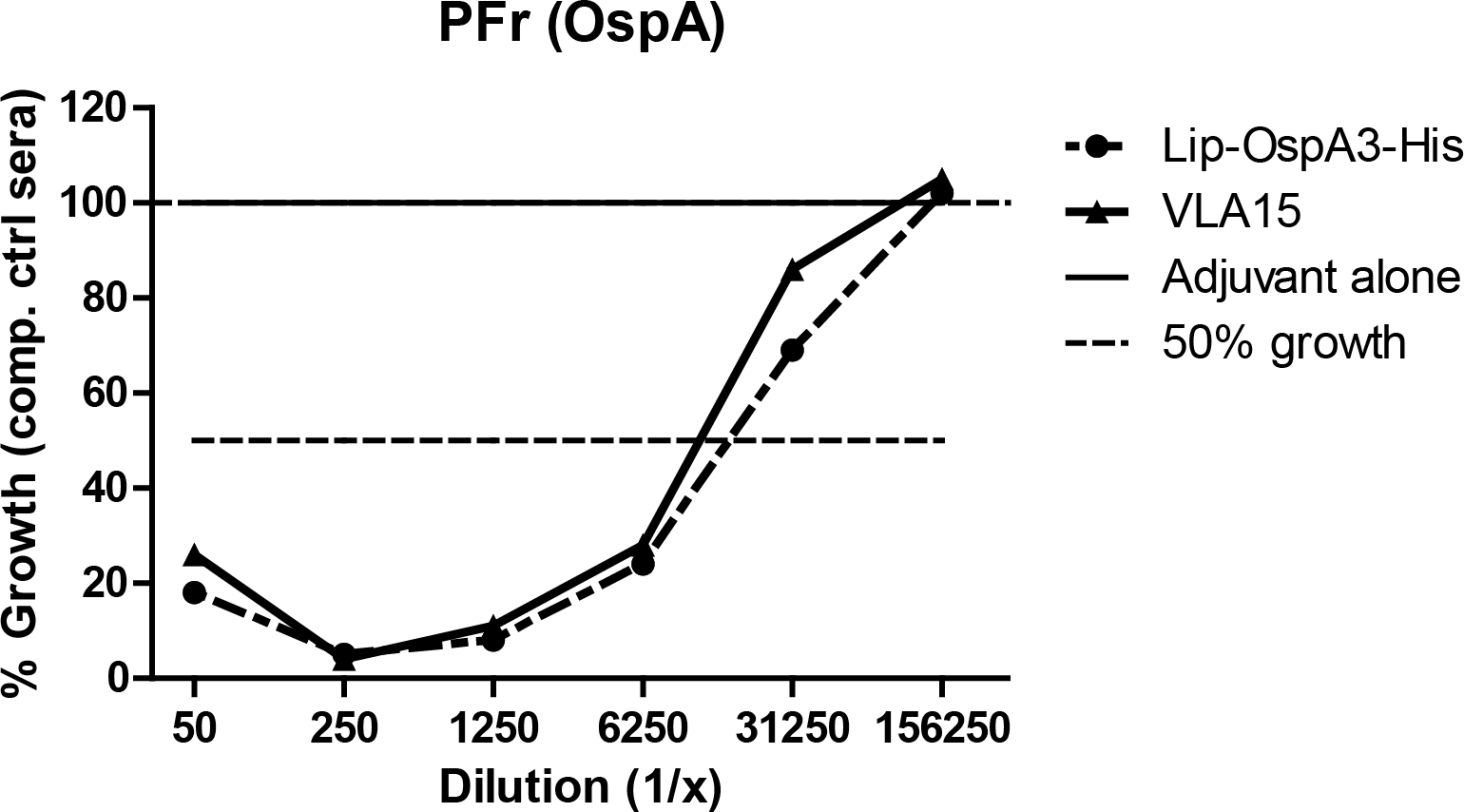
Growth inhibition of *B*. *garinii* (ST3). The growth inhibition of *B*. *garinii* (ST3) strain PFr in the presence of either immune sera from mice immunized with VLA15 or full length OspA (ST3, Lip-OspA3-His) are shown. The maximum level of growth, 100% (solid line), is defined as the growth observed in the presence of serum from placebo immunized mice. In addition, 50% spirochete growth is indicated (dotted line). VLA15 immune sera and full length OspA ST3 immune sera showed similar levels of growth inhibition.

We have previously described the difficulty in setting up a growth inhibition assay for *B*. *garinii* (ST3) [6]. The assay relies on immune sera and active complement for lysis or growth inhibition of the spirochetes. However, *B*. *garinii* (ST3) were sensitive to guinea pig complement alone, even in the absence of active immune sera. Guinea pigs are often used as a source for complement in serum bactericidal assays [14]. We have tested several other sources for complement (mouse, rabbit and human) without improving the performance of the assay. However, with the notion that *B*. *garinii* in general is more associated with birds than rodents in the enzootic cycle we sought to evaluate the use of avian complement for the growth inhibition assay. Interestingly, when chicken complement was used together with heat-inactivated immune sera from mice immunized with VLA15 or full length OspA (ST3, Lip-OspA3-His), the assay showed a growth inhibition titer for *B*. *garinii* (ST3) of 6,250 (Fig 1). Identical growth inhibition titers were measured for *B*. *bavariensis* (ST4) and *B*. *garinii* (ST5) in our earlier study, suggesting a similar potency of the immune sera against *B*. *garinii* ST3 as against ST4 and ST5 [6].

### VLA15 provides long term immunogenicity and can effectively be boosted in mice

**Fig 2:**
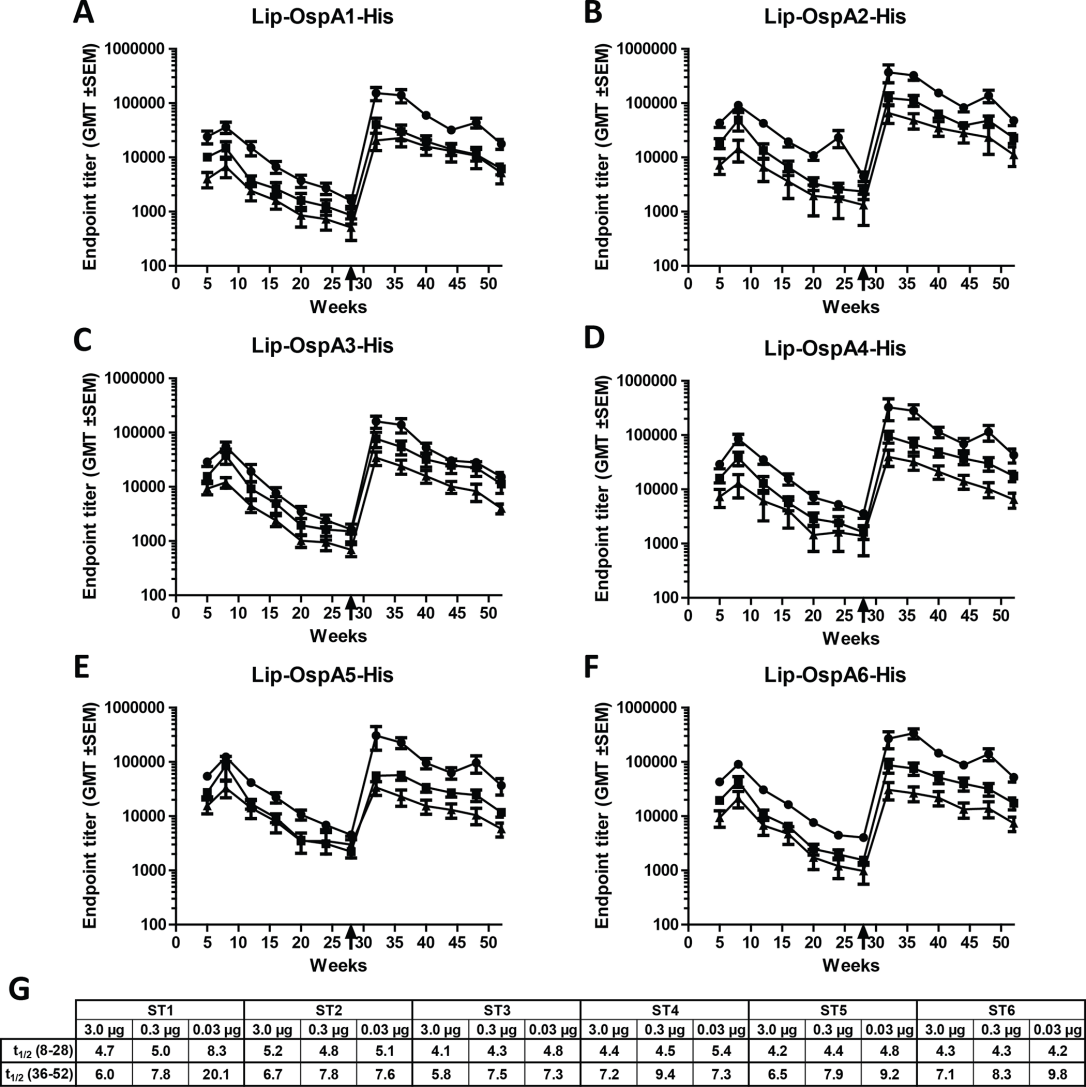
Long-term persistence of anti-OspA antibodies and the effect of a booster immunization. Mice were immunized subcutaneously three times with 3.0 **μ**g (-●-), 0.3 **μ**g (-■-) or 0.03 **μ**g (-▲-) VLA15 formulated with 0.15% aluminium hydroxide with two week intervals. Five months after the third priming immunization, a booster immunization was administered, indicated with an arrow on the time scale. Blood was collected monthly for one year and the total OspA serotype specific IgG titers determined with endpoint ELISA, where the plates were coated with full length OspA (Lip-OspA-His) of the corresponding serotype. (A) *B*. *burgdorferi* (ST1), (B) *B*. *afzelii* (ST2), (C) *B*. *garinii* (ST3), (D) *B*. *bavariensis* (ST4), (E) *B*. *garinii* (ST5), (F) *B*. *garinii* (ST6). (G) Anti-OspA antibody titer half-lives in weeks after primary immunization (week 8 to 28) and after booster immunization (week 36 to 52), respectively.

The long term persistence of anti-OspA serotype specific antibodies in mice immunized with VLA15 was assessed. Mice were immunized three times with 3.0, 0.3 or 0.03 μg VLA15 formulated with 0.15% aluminium hydroxide. Blood was collected every four weeks throughout the study and the immune response was measured with endpoint ELISA. The endpoint titers four weeks after the third immunization were ≥2.5×10^4^ (3.0 μg), ≥1.0×10^4^ (0.3 μg) and ≥3.5×10^3^ (0.03 μg) for all six serotypes (Fig 2). Following the priming immunizations, the antibody titers declined over time, with half-lives of approximately 4–5 weeks. A booster immunization was administered at five months after the third immunization, which resulted in a significant elevation of antibody titers. Following the booster immunization, the antibody titers declined over time with half-lives of approximately 7–8 weeks. The comparison of the six OspA serotypes revealed no significant differences regarding the immune response after the priming immunizations or the booster immunization. However, the anti-OspA ST1 antibodies in the lowest dose group (0.03 μg), declined with a much longer half-life than the antibodies against the other OspA serotypes (Fig 2).

## Discussion

The design of the described Lyme borreliosis (LB) vaccine relies on the protective capacity of the C-terminal fragment of OspA, using the sequence from the six serotypes most commonly associated with human infections. The six C-terminal fragments were linked together in pairs to form three fusion proteins, which have been described previously [6]. The fusion protein including the C-terminal fragments from OspA ST3 and ST4 was further optimized to improve immunogenicity and protein yields after expression and purification. The new fusion protein (Lip-D4Bva3B), where approximately the first 1/3 of the OspA ST3 sequence had been exchanged with the corresponding sequence from *B*. *valaisiana*, showed increased induction of anti-OspA ST3 specific immunogenicity and an overall yield in the same range as the other two heterodimers in the vaccine [8]. The new formulation including the improved fusion protein (Lip-D1B2B, Lip-D5B6B and Lip-D4Bva3B) of the LB vaccine candidate is referred to as VLA15. In this publication, we describe new challenge models with additional *Borrelia* species and demonstrate broad protection following active immunization with VLA15 also when administered at low doses. Furthermore, passive immunization with VLA15 induced antibodies showed broad protection in the same challenge models.

Earlier LB vaccines were developed for the North American market (LYMErix and ImuLyme) and therefore used only full length OspA ST1, providing protection against *B*. *burgdorferi* (ST1) infection [1,2]. However, the complicated situation in Europe, where several *Borrelia* species expressing different OspA serotypes cause LB, demands a different approach in order to develop a vaccine with broad protection. Efforts to develop a second generation OspA based LB-vaccine for the use in Europe and North America have recently been reported [3,26]. Although the multivalent chimeric OspA vaccine was designed to protect against infection with spirochetes expressing OspA ST1—ST6 [3,26], the preclinical testing has only demonstrated protection with one of the chimeric proteins (rOspA 1/2) against challenge with *in vitro* grown *B*. *burgdorferi* (ST1) or ticks harboring *B*. *afzelii* (ST2) [13,20]. Subsequently, immune sera obtained from the clinical trial with the multivalent OspA vaccine were demonstrated to bind to the surface of spirochetes expressing all six OspA serotypes included in the vaccine and to neutralize growth of spirochetes expressing five of the six OspA serotypes. However, growth inhibition activity against *B*. *garinii* (ST3) was not shown [3].

Here we show that immunization with VLA15 protects mice against challenge with 6 strains from four different *Borrelia* species; *B*. *burgdorferi*, *B*. *afzelii*, *B*. *bavariensis* and *B*. *garinii*, including in total five different OspA serotypes (ST1, ST2, ST4, ST5 and ST6). No other LB vaccine candidate, has thus far been shown to have a comparative broad range of protection in preclinical testing [7,11–13,20,22]. These results are very encouraging since the assessed *Borrelia* species together are responsible for the vast majority of human LB cases in Europe and the US [27–31]. In addition, the protective capacity of VLA15 was demonstrated primarily by challenge with infected ticks rather than using only *in vitro* grown spirochetes. The *Borrelia* isolates used for infecting ticks have never been passaged *in vitro*, but have only been maintained in a rodent-tick infection cycle in the laboratory to maintain plasmids and relevant virulence factors. Therefore, the challenge reflects as much as possible the natural situation, further verifying the protective capacity of the VLA15 vaccine.

Until now we have not been able to set up a challenge model with *B*. *garinii* (ST3), neither with *in vitro* grown spirochetes nor by establishing a colony of ticks infected with *B*. *garinii* (ST3). However, *B*. *garinii* (ST3) are believed to primarily rely on birds as reservoir hosts in nature [32,33], probably explaining the limited infectivity in mice and the sensitivity to rodent complement proteins as observed [34,35]. This likely also explains the very poor transfer to xenodiagnostic ticks and persistence during the subsequent molting process. The lack of a challenge model with *B*. *garinii* (ST3) was compensated though by the successful development of a reliable and robust growth inhibition assay with *B*. *garinii* (ST3). By using active complement from birds in the growth inhibition assay, we could demonstrate that VLA15 induces a functional immune response which also targets and neutralizes *B*. *garinii* (ST3).

The vaccine efficacy studies presented in this study, together with our earlier published data [6], show that VLA15 can induce a broad protective immune response targeting the majority of LB causing spirochetes in Europe and the US.

All OspA based vaccines rely on the neutralization of spirochetes by anti-OspA antibodies in the tick mid-gut before migration to the new host can occur [15]. Therefore, a successful vaccine needs to induce high and preferably long lasting OspA specific antibody titers. However, while LYMErix was formulated with aluminium hydroxide, ImuLyme did not include an adjuvant [1,2]. The unknown but possible need for relatively frequent booster immunizations was mentioned as one of several reasons why LYMErix was taken off the market [4,5]. We have earlier shown that including aluminium hydroxide in the VLA15 formulation increases the immunogenicity ten-fold in mice [6]. The need for aluminium hydroxide in the VAL15 formulation will be confirmed in toxicology studies as well as in clinical trials. The large increase in anti-OspA antibody titers following the booster immunization five months after primary immunization, indicates that VLA15 induces a strong immunological memory.

In summary, we can conclude that VLA15 has the potential to protect against the majority of clinically relevant *Borrelia* species causing LB in Europe, the US and possibly globally. Furthermore, the immune response is significantly enhanced following a booster immunization five months after the priming schedule. The safety and immunogenicity of VLA15 will therefore be further assessed in clinical trials.
